# Recent Advances in Metal Decorated Nanomaterials and Their Various Biological Applications: A Review

**DOI:** 10.3389/fchem.2020.00341

**Published:** 2020-05-19

**Authors:** Asim Ali Yaqoob, Hilal Ahmad, Tabassum Parveen, Akil Ahmad, Mohammad Oves, Iqbal M. I. Ismail, Huda A. Qari, Khalid Umar, Mohamad Nasir Mohamad Ibrahim

**Affiliations:** ^1^School of Chemical Sciences, Universiti Sains Malaysia, Pulau Pinang, Malaysia; ^2^Centre for Nanoscience and Nanotechnology, Jamia Millia Islamia, New Delhi, India; ^3^Department of Botany, Aligarh Muslim University, Aligarh, India; ^4^School of Industrial Technology, Universiti Sains Malaysia, Pulau Pinang, Malaysia; ^5^Center of Excellence in Environmental Studies, King Abdulaziz University, Jeddah, Saudi Arabia; ^6^Department of Chemistry, King Abdulaziz University, Jeddah, Saudi Arabia; ^7^Department of Biological Science, King Abdulaziz University, Jeddah, Saudi Arabia

**Keywords:** nanomaterials, metal oxide, biological application, therapeutics, tissue engineering

## Abstract

Nanoparticles (nanoparticles) have received much attention in biological application because of their unique physicochemical properties. The metal- and metal oxide–supported nanomaterials have shown significant therapeutic effect in medical science. The mechanisms related to the interaction of nanoparticles with animal and plant cells can be used to establish its significant role and to improve their activity in health and medical applications. Various attempts have been made to discuss the antibiotic resistance and antimicrobial activity of metal-supported nanoparticles. Despite all these developments, there is still a need to investigate their performance to overcome modern challenges. In this regard, the present review examines the role of various types of metal-supported nanomaterials in different areas such as antibacterial, antifungal, anticancer, and so on. Based on the significant ongoing research and applications, it is expected that metal-supported nanomaterials play an outstanding role not only in medical but also in other important areas.

## Introduction

Nanomaterials have found its way into different fields such as energy, environment, food industry, medicine, and so on (Mir et al., 2013; Khan et al., 2015; Sultana et al., 2015; Umar et al., 2016). Nanomaterials are still garnering attention at the scientific and commercial level (Xue et al., 2019). The properties of nanomaterials were found to be size-dependent, and this contributed to valuable chemical and physical properties. Recent developments have resulted in the improvement in the modeling and designing of different medical and biological tools and applications (Rathore et al., 2019; Yaqoob et al., 2019). The nanotechnology has garnered important commercial exploitation in the modern world. Nanomaterials have unique properties due to their small dimensions (1–100 nm). They have high electrical properties, high mechanical and thermal stability, high surface area, and high optical and magnetic properties (Khoshnevisan et al., 2019; Yaqoob et al., 2020a). These enhanced properties have enabled nanomaterials to be used in different fields including electrical, magnetic, optical, and electronic devices. Furthermore, some nanomaterials are engineered such as the modification occurring in case of titanium oxide, silver oxide, copper, zinc, and so on, with some other materials. Some of them are also found in different commercial production zones, such as production of sunscreens and stain-resistant clothing, and also used for investigative purposes in pharmaceuticals, diagnostic kits, imaging, magnetic resonance imaging (MRI), drug delivery, and in many more medical equipment and procedures (DaRocha et al., 2019; Ferreira et al., 2019; Silva and Rocha, 2019).

**Figure 1:**
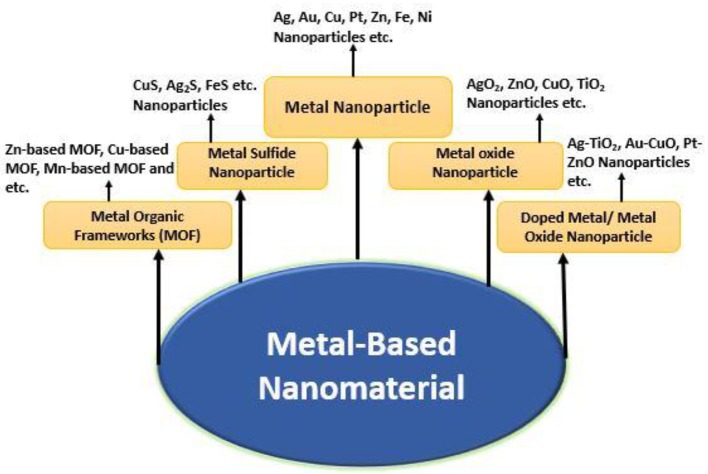
Different types of metal-based nanomaterials.

The nanomaterials that play a vital role in different applications in medical science commonly occur in some basic forms as shown in Figure 1. The first category of nanomaterial comprises the pure form of metal-based nanoparticles, which are also called metal nanoparticles (e.g., silver, copper, gold, titanium, platinum, zinc, magnesium, iron, and alginate nanoparticles). The other types of nanomaterials are the metal oxide nanoparticles (nanoparticles), that is, titanium dioxide, silver oxide, zinc oxide, and so on. The doped metal/metal oxide/metal nanomaterials are considered as another type of class among nanomaterials (Dar et al., 2011; Umar et al., 2013). Moreover, metal sulfide and metal organic frameworks (MOFs) nanomaterials have also concerned excellent interest because of their promising properties and applications in different biological fields. For example, AgS, CuS, FeS nanoparticles, Zn-based MOF, Cu-based, Mn-based MOF, and so on, are very commonly used in various medical applications such as drug delivery and antimicrobial activities.

Based on the nano concept, nanomedicine is also gaining more attention by making better diagnosis and developing different ways of treatment using nanoparticles in many diagnostic devices (Banerjee, 2018; Gurav et al., 2019; Ouyang et al., 2019). The noble metal nanoparticles (Ag, Au, Pt) have also attracted significant interest due to their photothermal and optical properties (Yaqoob et al., 2020b). These metal nanoparticles exhibit resonance electron oscillation known as localized surface plasmon resonance (Khan et al., 2017). The ability to integrate the noble metal nanoparticles into biological system with nontoxicity has impacted significantly medicinal and biological research. Among noble metallic nanoparticles, the gold nanoparticles got much attention due to its distinctive low toxicity, ease of preparation, and favorable attachment with biological molecules (Elahi et al., 2018).

The silver nanoparticles have been proven to be most useful because they have excellent antimicrobial properties against lethal viruses, microbes/germs, and other nucleus containing microorganisms. These nanoparticles are certainly the most extensively utilized material among all. Thus, it has been used as antimicrobial agent in different textile industries (Hasan, 2015). The noble metal nanoparticles are considered as more specific and multipurpose agents with a diversity of biomedical applications considering their use in extremely sensitive investigative assays, radiotherapy enhancement, gene delivery, thermal ablation, and drug delivery. These metallic nanoparticles are also considered to be nontoxic by the scientific community in case of gene and drug delivery applications. Moreover, metallic nanoparticles can offer diagnostic and therapeutic possibilities simultaneous (Yamada et al., 2015). Ceramics are another class of metal oxide nanoparticles that is currently emerging as antimicrobial agents for diagnostic purpose, in drug delivery devices, and agent in different pharmaceutical and medical treatments.

Some metal oxides also show antimicrobial activities, such as TiO_2_, Bi_2_O_3_, ZnO, FeO, MnO_2_, CuO, Ag_2_O, Al_2_O_3_, and so on, and play significant roles in various medical applications. For example, TiO_2_ has been used against the transmission of different infectious diseases (Janson et al., 2019; Mora et al., 2019). Similarly, ZnO nanoparticles are gaining worldwide attention due to their antibacterial activity. Zinc oxide is considered a biosafe material, and it is used to carry out photocatalysis and photo-oxidation reactions on biological and chemical species (Sirelkhatim et al., 2015). Moreover, Al_2_O_3_ nanoparticles have enormous viable applications and showed antimicrobial behavior (Swaminathan and Sharma, 2019). Sankar et al. (2014) reported that the CuO complex of *Ficus religiosa* nanoparticles was used as an anticancer agent in biomedicine because of its excellent chemical properties. The zinc-doped titania nanoparticles have revealed enhanced proangiogenic properties, which might be useful in different applications (Hadi et al., 2018). In another study, doped copper/TiO_2_ nanoparticles with carbon-based allotrope such as graphene oxide were reported as a new antimicrobial agent. The CuO has been utilized as an antimicrobial agent to degrade various microbial species (Nethi et al., 2017).

However, (Shivashankarappa and Sanjay, 2015) studied the biological approaches for the synthesis of cadmium sulfide (CdS) nanoparticles and used as antimicrobial agent against the diet borne pathogens. The antimicrobial activity results showed that the CdS nanoparticles have the highest zone of inhibition in case of *Aspergillus flavus* and *Pseudomonas aeruginosa*. Recently, (Aishwarya and Sanjay, 2020) studied the green synthesis of CdS nanoparticles from *Escherichia coli* and analyzed their toxic effect on cancer therapy. Antimicrobial studies of CdS nanoparticles were also done on the food basis pathogens, and toxicity was determined on musculus skin melanoma (B16F10) and humanoid epidermoid carcinoma (A431) cell appearance. So, CdS nanoparticles were found to be extremely active on cancerous cells as compared to normal anticancer drugs. Cai et al. (2019) studied the stimuli-responsive nanomaterials due to rapid growth of nanotechnology, which provided an alternate way for the designing of well-behaved drug delivery schemes due to their spatiotemporally well-behaved properties. Furthermore, the MOFs have also been broadly employed in medical applications, particularly in drug delivery, due to tunable hole size, large surface area and high pore volume, and informal surface variation. The main aim of this review is to explore the importance of metal-based nanomaterials (such as metal nanoparticles, metal oxide nanoparticles, doped metal/metal/metal-oxide, metal sulfide, and MOF) for their effective applications in biomedicine. This review also considers the role of nanoparticles in biology reported by many researchers along with their potential applications. Finally, a brief discussion is made to indicate the future perspectives of metallic nanomaterials.

## Role of Nanoparticles

Antibiotic-resistant pathogens have become a serious threat to human health. Among them, pathogenic microbes such as *Geobacter, Staphylococcus, Enterococcus*, and *Streptococcus* are responsible for various serious diseases and infections (Olishevska et al., 2019). These bacterial infections and diseases may lead to death of the affected individual at severe stage. New strategies are needed to prepare the advance level drugs or antibiotics to control these bacterial infections. Recently, nanotechnology has provided a great potential in several area of engineering, environmental science, medical science, and other fields.

Recently, the different types of metallic nanoparticles and their derivatives (such as silver, gold, copper, nickel, platinum, titanium, and zinc nanoparticles) received significant attention for their effective antimicrobial properties. Similarly, metal oxides and doped metal/metal composites such as silver oxide, copper oxide, calcium oxide, magnesium oxide, titanium dioxide, and zinc oxide have various unique properties, powerful potencies, and spectral activity and showed excellent antimicrobial ability (Dizaj et al., 2014). Furthermore, the physicochemical properties and morphology of nanomaterials have been proven for their antimicrobial activities. It was identified that the nano size metal particles carry the powerful bactericidal effect to resist the bacteria (Mohammadi et al., 2011; Fellahi et al., 2013; Besinis et al., 2014). The positive charge on the surface of metallic nanoparticles enables their binding ability toward negative charged bacteria surface, which may increase the bactericidal effect. The shape of the nanoparticle is also a very important factor because it also has a strong influence on the antimicrobial activity (Bera et al., 2014).

### Metal-Based Nanoparticles

The metal-based nanoparticles such as silver, gold, copper, iron, zinc, platinum, and so on, received much attention in medicine. Faraday (1857) showed the metal nanoparticles can exist in solution. Much later, Kumar et al. (2018) studied the color and morphology of metallic nanoparticles. Currently, nanoparticles can be synthesized and improved by modifying the chemical groups, which help to bind the antibodies. Noble metal nanoparticles (Ag, Au, Pt) have been used for several biomedical applications such as anticancer, radiotherapy enhancement, drug delivery, thermal ablation, antibacterial, diagnostic assays, antifungal, gene delivery, and many others. The noble metal nanoparticles have some unique properties that make it more valuable. Metal nanoparticles can be functionalized with a variety of functional groups, such as peptides, antibodies, RNA, and DNA, to target different cells along with potential biocompatible polymers, for example, polyethylene glycol (Fan et al., 2018). Some important nanoparticles along with their biological applications to target biological cells are summarized in Table 1.

**Table 1:** Different metal nanoparticles with their potential applications.

**No**.	**Nanomaterials**	**Targeted sites**	**Applications**	**References**
1	Platinum nanoparticles	Cancer cell	Toxicity evaluation	Gehrke et al., 2011
2	Gold/Ag nanoparticles	Cancer cell	Imaging therapy, photothermal therapy	Shi et al., 2014
3	Gold nanoparticles	Cancer cell	Radiosensitizer applications	Cho et al., 2009
4	Platinum nanoparticles	Cancer cell	Toxicity evaluation	Pelka et al., 2009
5	Gold nanoparticles	Cancer cell	Radiosensitizer applications	Roa et al., 2009
6	Silver nanoparticles	Skin	Skin penetration evaluation	Crosera et al., 2015
7	Platinum nanoparticles coated with polyvinyl alcohol	Brain	Toxicity evaluation	Asharani et al., 2010
8	Gold and iron oxide nanoparticles linked with glutathione	Cancer cell	Radiosensitizer applications	Kim et al., 2010
9	Silver nanoparticles	Antimicrobial agent	Toxicity evaluation	Samberg et al., 2012
10	Platinum nanoparticles	Cancer cell	Therapeutic evaluation	Porcel et al., 2010
11	Gold nanoparticles linked with glucose	Cancer cell	Radiosensitizer applications	Roa et al., 2012
12	Silver nanoparticles	Antimicrobial agent	Antimicrobial assessment	Sabella et al., 2011
13	Gold nanoparticles	Cancer: glioblastoma-based multiforme	Radiosensitizer applications	Joh et al., 2013; Sun et al., 2013
14	Silver nanoshell	Cancer cell	Photothermal ablation	Kleinauskas et al., 2013
15	Silver nanoparticles	Antimicrobial agent	Toxicity evaluation	Samberg et al., 2009
16	Silver nanoparticles capped with starch	Antimicrobial agent	Toxicity evaluation	Wang S. et al., 2019
17	Silver nanoparticles linked with polyvinylpyrrolidone	Brain cancer	Therapeutic evaluation	Locatelli et al., 2014
18	Silver nanoparticles	Wound healing	Therapeutic evaluation	Liu et al., 2010
19	Silica–gold nano shells	Cancer	Photothermal-based therapies	Pattani and Tunnell, 2012
20	Gold-branched shell nanostructures	Breast cancer	Imaging therapy, photothermal therapy, chemotherapeutic therapy	Topete et al., 2014
21	Superparamagnetic FeO nanoparticles coated with Si/Au nanoshells	Head and neck cancer	Photothermal therapies	Melancon et al., 2011
22	Silica–gold nanoshells	Brain tumor	Photothermal-based therapies	Choi et al., 2012
23	Magnetic nanoparticles	Not specific	Drug delivery, magnetic hyperthermia, MRI contrast agent, magnetic separation, controlled medicine release, cellular therapies	Estelrich et al., 2015
24	Magnetic nanoparticles	Cancer cell	Chemotherapies, biosensors, and imaging applications	Mohapatra and Anand, 2010
25	Magnetic nanoparticles	Nanofertilizers, nonfungicides, nanopesticides	Nanosensors, nanocoatings, nanocomposites, food packing, remote-sensing devices, gene transfer, etc.	Srivastava, 2014
26	Magnetic nanoparticles	Wastewater	Wastewater treatment	Xu et al., 2012
27	Silica–gold nanoshells	Cancer	Photothermal based therapies	Trinidad et al., 2014
28	Au nanoparticles	Intravascular tissue	Therapeutic agents	Giasuddin et al., 2012
29	Tiny Au nanoparticles	Tiny Au nanoparticles	Anticancer therapies	Giasuddin et al., 2012
30	Not specific	Au nanoparticles	Different types of surgical devices applications	Giasuddin et al., 2012

Xiong et al. (2011) synthesized copper nanoparticles with less than 2 nm particle size by using l-ascorbic acid. It was used as a stabilizer and as a reducing agent. These nanoparticles were used as an efficient antibacterial agent against gram-negative and gram-positive bacteria. (Tomar and Garg, 2013) prepared the Au nanoparticles for identification of protein interactions and used it in the detection and evaluation of DNA from biological samples. Gold nanoparticles have been used as a detector in aminoglycoside antibiotics, to identify the cancer cells, and also it was found useful in identification of different microorganisms. Dreaden et al. (2012) used Au nanoparticles in cancer diagnosis and its imaging through transporting the nanoparticles into targeted cell nucleus. This highlighted the role of nanoparticles in different biomedicine applications.

The health care industries are facing challenges due to antimicrobial resistance by pathogenic bacteria. Rai et al. (2009) studied the Ag nanoparticles and checked the combined effect with antimicrobial activities. The antimicrobial activity of amoxicillin, penicillin G, clindamycin, vancomycin, and erythromycin were found to be improved owing to the presence of Ag nanoparticles against the strains. Keat et al. (2015) elaborated the role of Ag nanoparticles in biomedicine to serve as cell imaging, cancer therapy, genetic delivery, drug delivery, and different disease diagnosis. Ag nanoparticles have greatly influenced the medical practice, as well as the health care unit. Fellahi et al. (2013) observed the antibacterial activities of silicon nano substrates linked with Ag nanoparticles or Cu nanoparticles. Their study concluded that the synthesized nanoparticles have improved antibacterial activities against *E. coli*. The Ag-coated silicon wires were biocompatible with regard to the human lung, especially adenocarcinoma epithelial cells, whereas the Cu-coated Si nanowires exhibited high cytotoxicity that may lead to death. Siddiqi and Husen (2017) studied the medical applications of Pd nanoparticles and tried to make them more prolific. Pd nanoparticles can act as anticancer and stabilizing agents in many pharmaceutical products.

### Metal Oxide–Based Nanoparticle

The role of metal oxide nanomaterials has been studied with excellent results in biomedicine. Metal oxide nanoparticles such as Ag_2_O, FeO, MnO_2_, CuO, Bi_2_O_3_, ZnO, MgO, TiO_2_, CaO, Al_2_O_3_, and many others were acknowledged to show potential antibacterial activity. Some reported metal oxides used in biomedical application are listed in Table 2.

**Table 2:** Role of different metal oxides in biomedical field.

**Sr no**.	**Nanomaterial**	**Applications**	**References**
1	Iron oxide	Magnetic imaging, environmental remediation applications	Schrand et al., 2010
2	Silver oxide	Antimicrobial, drug delivery, gene therapies, tissue developments, imaging, etc.	Shanmuganathan et al., 2019
3	Cerium oxide	Bioimaging and surgical devices, etc.	Schrand et al., 2010
4	Silica oxide	Production of thermal and electric insulators gene delivery, catalyst applications, drug carriers, efficient adsorbents, serve as filler materials, etc.	Schrand et al., 2010
5	Zinc oxide	Skin protectant, etc.	Pantic et al., 2019
6	Titanium dioxide	Antimicrobial, coating material, sterilization	Esmaeilnejad et al., 2019
7	Nickel (oxide)	Biomedical applications such as anticancer	Khan et al., 2019
8	Copper oxide	CuO can work as, antibiotic, antiviral, antimicrobial, antifouling, and antifungal treatment and many other nonmedical applications such as inks, coating materials, catalyst factor, lubricants, filler substance for enhanced wear resistance and conductivity	Schrand et al., 2010
9	Gold oxide	Antimicrobial, drug delivery, cellular imaging, photodynamic therapies, cancer treatment, surgical devices, etc.	Schrand et al., 2010
10	Aluminum oxide	Antifungal, antibacterial, antiviral, etc.	Subramaniam et al., 2019
11	Magnetic iron oxide	Drug delivery, tissue repairing, cellular labeling, hyperthermia, etc.	Tong et al., 2019
12	Calcium oxide	Strong antimicrobial activity connected to active oxygen species and alkalinity	Dizaj et al., 2014
13	Magnesium oxide	Antibacterial applications	Dizaj et al., 2014
14	Bismuth oxide	Drugs delivery systems	Szostak et al., 2019
15	Chromium oxide	Used in improving collagen stability	Sangeetha et al., 2012
16	Manganese dioxide	Biocatalysis, fluorescence sensing, controlled drug delivery, stimuli-activated imaging	Wu et al., 2019

Sathyanarayanan et al. (2013) studied the impact of Ag_2_O nanoparticles and considered it as a novel source of antibiotics. Furthermore, Salas et al. (2019) also demonstrated the antimicrobial properties of Ag_2_O nanoparticles against *E. coli*. The ZnO nanoparticles exhibited good bactericidal effects against the gram-positive and gram-negative microorganisms and spores. These microorganisms and spores are not affected by high pressure and temperature. Moreover, Prasanna et al. (2019) explored the antibacterial activities of ZnO with different particle sizes. From the results, it was proved that the bactericidal efficacy of ZnO nanoparticles increased as the particle size decreased. Azam et al. (2012) studied the relative antimicrobial activities of CuO, ZnO, and Fe_2_O_3_ nanoparticles effect on gram-negative bacteria, such as *E. coli, P. aeruginosa*, and so on, and gram-positive bacteria, such as *Staphylococcus aureus* and *Bacillus subtilis*. From these results, ZnO was found to have a good antimicrobial activity, whereas Fe_2_O_3_ nanoparticles showed the least antibacterial effect.

The antibacterial activity of TiO_2_ nanoparticles is associated to its crystal structure and its morphology, such as size, shape, and so on. Roy et al. (2010) discussed the TiO_2_ nanoparticles effect with diverse antiagents against methicillin-resistant *S. aureus*. TiO_2_ nanoparticles enhanced the antimicroorganism effect of β-glycopeptides, lactums, macrolids, cephalosporins, aminoglycosides, and tetracycline against methicillin-resistant *S. aureus*. Haghighi et al. (2013) studied the antifungal effect of TiO_2_ nanoparticles on biofilm of fungus. The results demonstrated that the synthesized TiO_2_ nanoparticles had enhanced antifungal properties on *Candida albicans* biofilms. The authors also suggested that TiO_2_ nanoparticles could efficiently hinder the fungal growth on biofilms, particularly those produced on medical devices. Another challenge of TiO_2_ nanoparticles is its toxicity, but the doping and conjugation with nontoxic material were used and proved to be a very novel approach. Similarly, MnO_2_, Bi_2_O_3_, and FeO got significant attraction in the field of biomedical such as drug delivery, bioimaging, antimicrobial activities, and many more. For example, Wang Y. et al. (2017) reported the preparation of bovine serum albumin–functionalized FeO nanoparticles (4.8 nm) with higher relativity value, that is, 444.56 mM^−1^ s^−1^, which can serve as *in vivo* bioimaging of tumor cells. Similarly, in another study, Gao et al. (2018) introduced a theranostic-based nanocomposite with multiscale FeO nanoparticles for magnetic resonance bioimaging and chemotherapy for treatment of breast cancer. Similarly, MnO_2_ is considered as an ideal compound because of having remarkable physicochemical, structural, and morphology-based properties. The remarkable 2D planar structure and of MnO_2_ nanoparticles are very significant for medical applications (Wu et al., 2019). Chen et al. (2019) studied the MnO_2_ nanosheets and explored it for various applications such as biological imaging, biosensing, cancer therapy, molecular adsorption, and drug delivery. The outcomes showed that the MnO_2_ nanosheets show less cytotoxicity and higher hemo/histocompatibility. Ovsyannikov et al. (2015) synthesized the spherical Bi_2_O_3_ nanoparticles (35 nm) and used as cancer cell aggressive agent. It was recommended to use a mixture of achieved nanospheres with phenothiazine photosensitizer, which showed drug delivery properties. Szostak et al. (2019) studied the Bi_2_O_3_ nanoparticles and successfully used as drug carrier in medical field. Ahamed et al. (2014) revealed that the CuO nanoparticles had substantial antimicrobial properties against different bacterial strains such as *Proteus vulgaris, E. coli, Klebsiella pneumoniae, Shigella flexneri, P. aeruginosa, Enterococcus faecalis, Salmonella typhimurium*, and *S. aureus*. Among these, *E. faecalis* and *E. coli* presented maximum sensitivity to CuO nanoparticles, whereas *K. pneumoniae* was unaffected by the nano formulations. The CaO nanoparticles and MgO nanoparticles show good antibacterial activities in an alkaline environment and oxygen. Leung et al. (2014) synthesized the MgO nanoparticles and studied its antibacterial activities. They showed that the route may be due to damage of the cell membrane. The MgO nanoparticles was shown to have antimicrobial effect on gram-positive and gram-negative bacteria. Leung et al. (2016) studied the antibacterial properties of MgO nanoparticles against *E. coli* and *S. aureus*. The authors recommended that the active oxygen on MgO nanoparticles was key the factor contributing to the bacterial activities. The MgO and CaO nanoparticles show excellent antibacterial properties. It can be prepared at low cost with easily available materials and have excellent biocompatibility. These materials can also be used in food processing and environmental preservation besides their biomedical uses.

### Doped Metal/Metal/Metal Oxide–Based Nanoparticles

Researchers are now focusing on the modification of nanomaterial to make it more stable during the chemical processing and to make the materials safe for the ecosystem. The doped metal/metal and metal/metal oxide nanoparticles are known to lead to enhanced efficiency in the biomedical applications of metal oxides along with their major outcomes. These are summarized in Table 3.

**Table 3:** Doped nanomaterials with their major outcomes.

**S. no**.	**Nanomaterial**	**Dopant**	**Application**	**Major outcomes**	**References**
1	ZnO	Co	Antimicrobial activity	ZnO doped with Co nanoparticles was identified to be crystalline with a single phase - It increased crystallite size from 20.5 nm to 25.7 nm - Doping enhanced the antibacterial activities of composite to control marine borne pathogen	Oves et al., 2015
2	ZnO	TiO_2_	Water decontamination applications, angiogenic applications	- Doped composite is found good agents for multifunctional environmental applications - Zn-doped titania nanoparticles composite have exposed enhanced proangiogenic properties	Nethi et al., 2017
3	Fe_3_O_4_	Gelatin	Drug delivery, MRI, different therapies, fluorescence sensor, etc.	- Enhanced the biomedical application efficiency in different zones	Cheng et al., 2014
4	Polycrystalline ZnO	Mn	Antimicrobial activity	- Result demonstrated that the Mn-doped ZnO nanoparticles increased antibacterial activities than pure ZnO nanoparticles	Rekha et al., 2010
5	Ag	Zn	Antimicrobial applications	- Doped composite enhances the performance against *E. coli* and Vibriocholerae	Salem et al., 2015
6	TiO_2_	ZnO/graphene oxide	Drug delivery	- They showed the substantial toxicity; due to this, the cell viability condensed	Zamani et al., 2018
7	ZnO	Fe	Cytotoxicity screening applications	- This doping used to enhance the nanosafety by reducing ZnO through doping	George et al., 2009
8	ZnO	Ta	Antibacterial applications	Ta-doped ZnO nanoparticles composite showed more active bactericidal value than pure ZnO in presence of dark ambient and improved the synergistic effect with surface bioactivity	Guo et al., 2015
9	ZnO	TiO_2_	Biomedical applications	- The results showed that the nanoparticles composite can be genotoxic without being cytotoxic	Osman et al., 2010
10	TiO_2_	Reduced graphene oxide	Ambient light-based antimicrobial activities		Dhanasekar et al., 2018
11	TiO_2_	Cu	Ambient light-based antimicrobial activities	- Doping with Cu@ TiO_2_ promoted degradation of different microorganisms	Dhanasekar et al., 2018
12	Zn	CuO	Multidrug-resistant bacteria applications	- Mechanism of antibacterial activity is enhanced	Malka et al., 2013
13	TiO_2_	Ag_2_O	Drug-resistant bacterial applications	- Doped material enhanced the resistant ability against leishmania parasites	Allahverdiyev et al., 2011
14	Multiwalled CNT	Ag	Biomedical applications	- Used for cellular viability and cellular proliferation	Madhumitha et al., 2015
15	Zirconium titanium phosphate	Ag	Antibacterial applications	Found best antibacterial agent against *E. coli*	Biswal et al., 2011

The ZnO doped with Sb or Mg may increase the antibacterial activities of ZnO nanoparticles. The doped metallic nanoparticles were recommended to be used in different applications in pharmaceuticals because it has less self-toxicity issues. Guo et al. (2015) studied the antimicrobial effect of tantalum-doped zinc oxide nanoparticles on numerous microbes of gram-positive bacteria such as *B. subtilis, S. aureus*, and gram-negative *E. coli* and *P. aeruginosa*. The Ta- and ZnO-doped nanoparticles showed more active antimicrobial capability than ZnO nanoparticles in ambient darkness. It increased the surface bioactivities and enhanced the electrostatic force due to the combination of Ta^5+^ ions with ZnO nanoparticles. The results revealed enhancement in antibacterial activities by using 5% doped nanoparticles than other pure metal oxide nanoparticles.

Malka et al. (2013) synthesized the Zn-doped CuO and deposited in on the surface of cotton fabric through ultrasound irradiation. A colloidal suspension of Zn-doped CuO was used to deposit on the surface of the cotton fabric. The antimicrobial activities of this treated fabric were used against gram-negative and gram-positive bacteria. A significant improvement of 10,000 times was observed in the antibacterial activity of Zn-CuO–doped nanoparticles compared to pure CuO nanoparticles and ZnO nanoparticles. Rekha et al. (2010) used doped Mn/ZnO nanoparticles to study the photocatalytic and antibacterial activity. The effect of Mn doping was studied with respect to doping concentration, its structural morphology, and optical properties. The results showed that Mn/ZnO nanoparticles increased the antibacterial activities compared to the metal oxide (ZnO) nanoparticles.

Dhanasekar et al. (2018) introduced the Cu-doped TiO_2_ nanoparticles in the presence of reduced graphene oxide (rGO), which served as the solid support. It was then used as an innovative light antibacterial agent. The results suggested that the nanoparticles of Cu_2_O-TiO_2_ in the presence of rGO showed improved visible light antimicroorganism properties with advanced inhibition zone and lesser value of least inhibitory concentration for gram-positive and gram-negative microorganisms as compared to pure TiO_2_.

Llorens et al. (2012) stated that Ag-doped MgO showed significant important antimicrobial activities in biomedical applications. Nganga et al. (2013) elaborated the Ag polysaccharide doping with fiber reinforcement composites. It was exposed for antimicrobial activities against *S. aureus* and *P. aeruginosa*. The Ag-fiber reinforcement composites presented excellent antimicrobial efficiency against microbial strains. Arakawa et al. (2019) studied the antimicrobial activity of Ag and carbon monolith doped nanoparticles. Carbon monolith with Ag doping also showed improved antimicrobial efficiency against *C. albicans, S. aureus*, and *E. coli*. Furthermore, Ag doped with phosphate-based glasses was investigated by Zhu et al. (2019) to explore the antimicrobial activities through disk diffusion analyses against certain disease-causing pathogens such as *S. aureus, E. coli, Bacillus cereus, P. aeruginosa*, and so on. Ewald et al. (2011) used the Ag from cements against *S. aureus* and *Staphylococcus epidermidis* growth. The results showed excellent antibacterial properties.

### Metal Sulfide–Based Nanoparticles

Metal-based chalcogenides are considered as the most promising semiconductor resource with many medical applications due to the presence of definite properties at nanometric levels. The included properties are high fluorescence; fine optical band gap; and excellent magnetic, thermal, mechanical, and structural stability (Vena et al., 2016). Furthermore, Quantum dots, nanocrystals, and metal-based chalcogenide nanoparticles have been projected for biomaterials in biosensor, drug delivery, biolabeling, bioimaging, and diagnostic purpose. There are some metal-based chalcogenides such as CdSe, PbS, CdSe-CdTe, CdSe-ZnTe, CdTe-CdSe, and so on, which have very complex structures (Li and Wong, 2017). Recently, metallic sulfides that contain chalcogenide sulfur are significantly bonded with a toxic-free metal, and they obtained much interest in the medical field (Dahoumane et al., 2016). Among them, the most common metal sulfides are AgS, CuS, FeS, and ZnS and are considered as the most important materials for biomedical applications.

**Figure 2:**
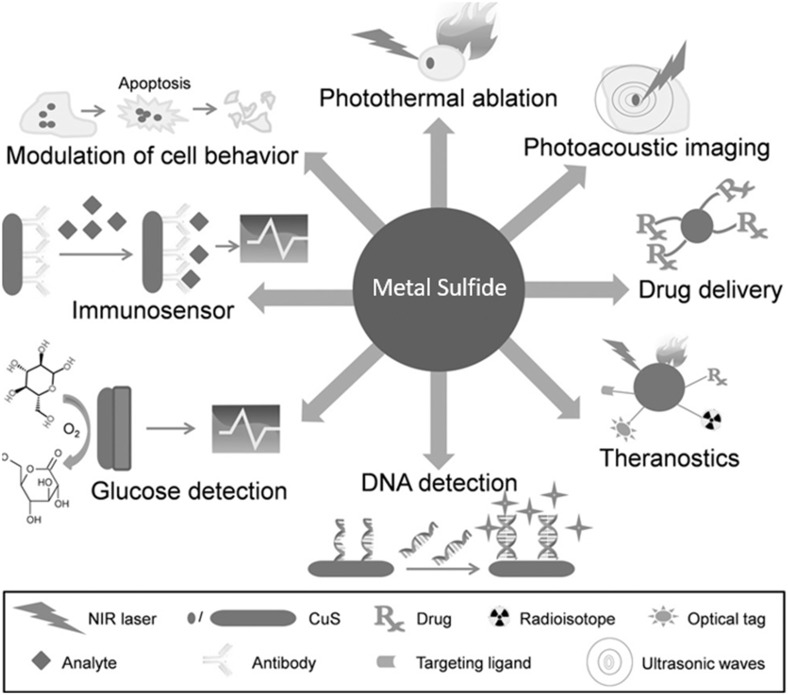
Potential medical applications of metal Sulfide based nanoparticles (CuS nanoparticles). Adapted from Goel et al. (2014), with permission from © Wiley@VCH Verlag GmbH & Co. K GaA, Weinheim.

However, Goel et al. (2014) studied the CuS nanoparticles, which are progressively developing as a most promising stage for biosensing, photothermal therapy, biomolecular imaging, and drug deliveries. The CuS nanoparticles showed significant role *in vitro* and *in vivo* applications. CuS nanoparticles and their derivatives have been extensively used in molecules detection technology such as DNA detections, metabolites (glucose) detections, food-based pathogen, and so on. The growing popularity of CuS nanoparticles in the field of biosensing is primarily based on conductivity and capacity of these nanoparticles, which encourage electron transfer reactions with molecules. Furthermore, CuS thin film–based immunosensor was introduced for the exposure of anthropological immunoglobulin A (IgA) antibody in serum, in which a goat antihuman IgA antimediator was immobilized on surface of CuS thin film (Attarde and Pandit, 2020). The functionalized quantum dots of Ag_2_S are also important to use in bioimaging and diagnostics purposes. The imaging, labeling, tissue imaging, diagnosis, and photodynamic treatment are few bioapplications of Ag_2_S. Another stimulating application of Ag_2_S quantum dots is tracking and designing of cells *in vivo*. Conventionally, proteins and dyes were considered as imaging tracking agent to achieve nanometer precision. Recent developments of metal sulfide–based nanoparticles (CuS nanoparticles) have covered an extensive variety of medical applications as shown in Figure 2.

The literature also showed that the Ag_2_S quantum dots can be used as an excellent active tracker for human mesenchymal stem cells *in vivo* within a range of near-infrared (1,000–1,400 nm). Ag_2_S nanoparticles were found as an excellent material to demonstrate the microbial progress inhibition. This is also a perspective application of Ag_2_S nanoparticles (Argueta-Figueroa et al., 2017). However, Kumari et al. (2014) found that the Ag_2_S nanoparticles show a reasonable antimicrobial consequence too. The Ag_2_S nanoparticles (0.1 μg/mL) treatment offered 75% growth inhibition rate against *E. coli* and *S. aureus* strains. Tian et al. (2011) reported the CuS nanoparticles as photothermal agents for the treatment of cancerous cells, although the 500 nm particle size limits their scientific version. Ding et al. (2016) synthesized the Fe_3_S_4_ nanoparticles along with pseudoenzyme activities. These activities were applied to enterprise a measurable photometric enzyme glucose and assess in human serum, which leads to a final product from an enzyme material that is oxidized by hydrogen peroxide through Fe_3_S_4_ nanoparticles. Similarly, Argueta-Figueroa et al. (2017) studied the Ag_2_S, CuS, and FeS significant value in the field of biomedical. Moreover, the summary of metal sulfide nanoparticle and their biomedical applications are shown in Table 4.

**Table 4:** Summary of metal sulfide nanoparticle and their biomedical applications.

**Sr. no**.	**Type of metal sulfide**	**Particle size (nm)**	**Applications**	**References**
1	Ag_2_S spherical nanoparticles	25	Antimicrobial activities	Ayodhya and Veerabhadram, 2016
2	CuS nanoparticles	11	Diagnostics applications through photoacoustic tomography	Ku et al., 2012
3	Au/CuS core/shell nanoparticles	5	Cancer treatment	Lakshmanan et al., 2012
4	Ag_2_S quantum dots	18	Analysis, therapy, and real-time bioimaging for modified treatment of tumor cells	Hu et al., 2015
5	Au/CuS core/shell nanoparticles	5	Antimicrobial activities	Addae et al., 2014
6	Ag_2_S nanoparticles	40	Photothermal transducing mediators for treatment of cancer	Ma et al., 2016
7	Fe_3_S_4_/Ag composite particles	–	Antimicrobial activities	He et al., 2014
8	Fe_3_S_4_ nanoparticles/citrate	32	Photothermal activities	Simeonidis et al., 2016
9	Ag_2_S spherical nanoparticles	30	Antimicrobial activities	Kumari et al., 2014
10	Flower-shaped CuS nanoparticles	50	Photothermal mediator for the ablation of cancerous cells	Tian et al., 2011
11	Ag_2_S quantum dots	5.4	*In vivo* imaging for fast tumor cell detection.	Hong et al., 2012
12	Thioglycolic acid–CuS nanoparticles	3	Photothermal therapy	Li Y. et al., 2010
13	Ag_2_S quantum dots chitosan nanosphere-based S-nitrosothiol	117	Cell fluorescence bioimaging for medical applications	Tan et al., 2013
14	ZnS nanoparticles	100	Antifungal activities	Suyana et al., 2014
15	CuS-reduced graphene oxide nanocomposite	400	Detection of hydrogen peroxide unconfined from living cells	Bai and Jiang, 2013
16	Fe_3_S_4_ magnetic nanoparticles	76	Glucose detection	Ding et al., 2016
17	ZnS nanoparticles	162	Antimicrobial activities	Li G. et al., 2010
18	Single-layer MoS_2_ nanoparticles	800	Chemotherapy for cancerous cells	Chou et al., 2013
19	ZnS nanoparticles	65	Antimicrobial activities	Malarkodi et al., 2014
20	Dendrimer-Bi_2_S_3_ nanoparticles	5	Bioimaging	Fang et al., 2013

### Metal Organic Frameworks

**Figure 3:**
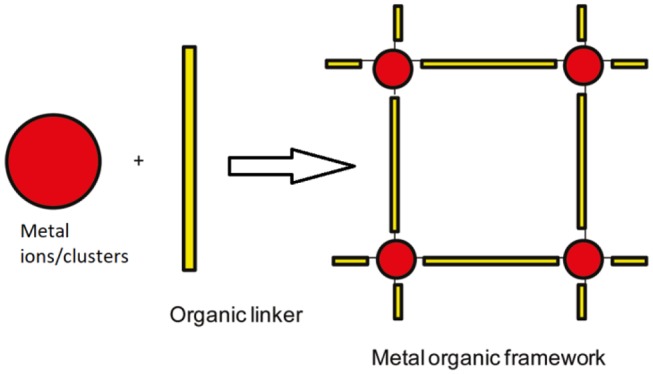
Formation of MOFs.

Metal organic frameworks are also known as a comparatively novel crystalline nanoporous material. The MOFs are defined as the self-assembly of metallic ions that serve as coordination centers and organic/inorganic–based ligands, which serve as linkers in metallic centers as shown in Figure 3 (Keskin and Kizilel, 2011).

**Figure 4:**
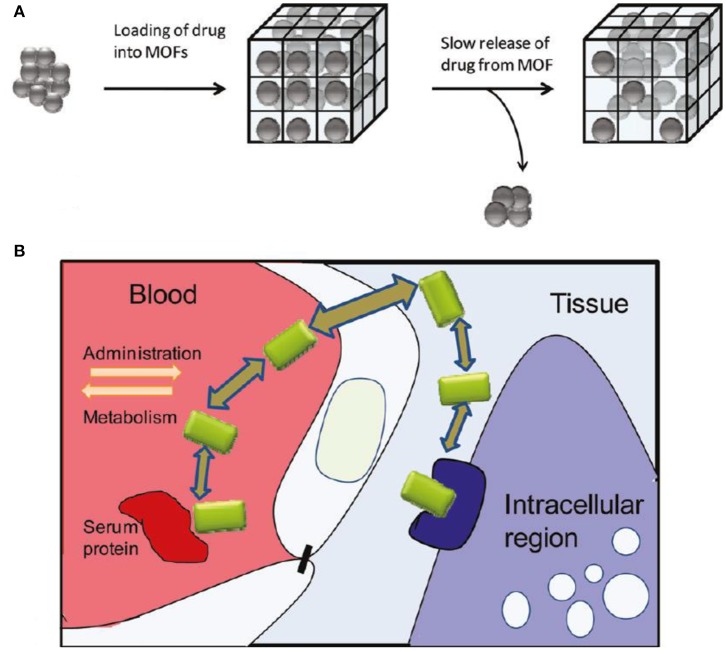
**(A)** Metal organic frameworks comprehensive scheme as drug delivery vehicles. **(B)** *In vivo* conditions elaborated in the deliberate discharge of drugs. Adapted with permission from Keskin and Kizilel (2011).

This is one of the most stimulating new expansions in nanoporous science, also known as porous coordination, hybrid organic-inorganic coordination networks. The MOFs have some unique properties that make it more prolific such as high porosity and high surface area, which are usually from 1,000 to 10,000 m^2^/g, which is higher than conventional porous materials (Arenas-Vivo et al., 2019). Metal organic frameworks extensively explored for drug delivery system in modern development due to excellent drug-loading capacity, informal functionalization, ideal biodegradability, and good biocompatibility. Keskin and Kizilel (2011) regarded the MOFs as optimal material for drug delivery application due to modification of pore size and some prospect of regulating functional groups. The MOF significant generalized system as drug delivery is shown in Figure 4 along with *in vivo* conditions.

An et al. (2012) studied the Zn-based MOFs to load the drugs and discharge for immobilization of larger molecules. Bio-MOF-100 named nanomaterial was effectively used for drug storage previously. Similarly, Briones et al. (2016) also studied the Zn ion bonded with some other materials, for use in antidiabetic activities. Hidalgo et al. (2017) studied the Mg metallic ion to achieve the Mg(H_4_gal) MOF material, which was effectively used against pathogens and used as antioxidant carrier too. The Ag metal ions also got significant attention due to their remarkable properties especially as antimicrobial agent. Arenas-Vivo et al. (2019) studied the Ag-based MOF material and used against bacterial diseases and achieved significant results. There are some combinations of metals and related linker as shown in Table 5, which worked perfectly for several types of biomedical applications.

**Table 5:** Combinations of metals/related linker and their role in various biomedical applications.

**Sr. no**.	**Metal ion**	**Organic linker**	**MOFs**	**Potential applications**	**References**
1	Zn	2-Methylimidazole	ZIF-8	Drug delivery (doxorubicin drug)	Vasconcelos et al., 2012
2	Zr	2-Aminoterephthalic acid	UiO-66	Drug delivery (drug caffeine)	Nagata et al., 2015
3	Zn	1,1′,4′,1″,4″,1-Quaterphenyl-3,5,3,5-tetracarboxylic acid 1,3,5-benzenetrisbenzoate	(NH_2_(CH_3_)_2_[Zn_3_(L)_2_ · 3.5DMF])	Drug delivery	Li Q. L., et al., 2015
4	Cu	[1,1′;3′,1″] terphenyl-4,5′,4″-tricarboxylic acid) and pyrazine	[Cu_3_L_2_(pyrazine) (H_2_O)]	Drug delivery of ibuprofen	Wei et al., 2016
5	Cu	5-NH_2_-m-benzenedicarboxylate	MOP-15	Drug delivery	Wang et al., 2011
6	Fe^3+^	2-Amino terephthalic acid	Fe-MIL-53-NH2-FA-5-FAM/5-FU	MRI and optical bioimaging	Gao et al., 2017
7	Gd^3+^	4-Dimethylaminopyridine	Gd-DTPA-FITC-CS11	Bioimaging	Wang et al., 2016
8	Zn	2-Methylimidazole	Fe_3_O_4_@PAA/AuNCs/ZIF-8 nanoparticles	MRI and CT bioimaging	Bian et al., 2015
9	Gd^3+^	Ru[4,4′-(COOH)2bipyridyl(bpy)]3	Gd-Ru MOFs	MRI and optical bioimaging	Huang et al., 2016
10	Fe^3+^	Fumarate	Au@MIL-88(A)	CT bioimaging	Shang et al., 2017
11	Cu	H3btc	Cu-BTC(MOF-199)	Antibacterial application	Rodríguez et al., 2014
12	Co	H8 tdm: tetrakis [(3,5-dicarboxyphenyl)-oxamethyl] methane	Co-TDM	Highly effective bactericidal activities application	Zhuang et al., 2012
13	Ag	HO-H_2_ipa = 5-hydroxyisophthalic acid and H_2_pydc = pyridine-3, 5-dicarboxylic acid	Ag_2_(O-IPA)(H_2_O)·(H_3_O) and Ag_5_(PYDC)_2_(OH)	Antibacterial application	Indumathy et al., 2007
14	Zn	—	Bio-MOF-100	Drug loading and release for immobilization of biomolecules	An et al., 2012
15	Mg	H_4_gal	Mg(H_4_gal)	Antioxidant carrier application	Hidalgo et al., 2017
16	Zn	AzA: azelaic acid	BioMIL-5	Antibacterial application	Tamames-Tabar et al., 2015

## Surface Modification Strategies to Improve Properties of Metal-Based Nanoparticles

**Figure 5:**
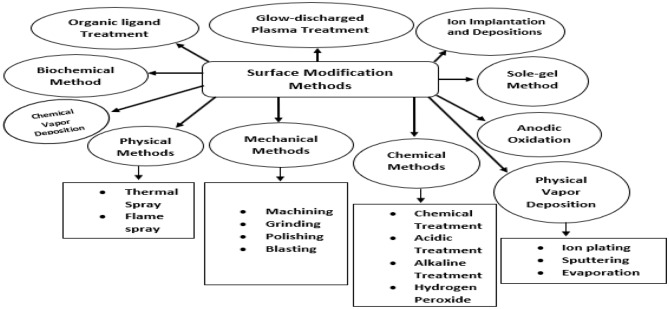
Summary of surface modification methods of metal-based nanoparticles.

The surface properties of new synthesized materials are usually insufficient in terms of biocompatibility, toxicity, adhesion properties, and wettability. Therefore, they should be improved up to sufficient extent prior to any kind of practical application or any potential processing technique such as coating with targeted materials. However, research on metal-based nanoparticles is growing rapidly, and several applications are predicted. Although many kinds of nanomaterials have outstanding physiochemical bulk features, they do not have appropriate surface specificity for special applications. Therefore, it may be essential to upgrade the surface properties. The surface modification of metal-based nanoparticles carried out with some significant advantages. First, the modification provides opportunity to stabilize nanoparticles against agglomeration. Second, it helps to empower their self-organization, and third, it creates interest to offer compatibility with others (Viswanathan et al., 2019). For example, when metal nanoparticles are attached with any suitable functional group, it can be made water-soluble. One more example is modification of inorganic (nano-)fillers with organic compounds. The surface modification can evade compatibility and homogeneity difficulties between the nano fillers and organic compounds and thus expand mechanical properties of organic/inorganic composite (Rahman et al., 2002). The surface modification of material that was used as medical kits is also needed to be modified before the treatment process. The clinical advantages achieved after surface modification are mentioned as good antimicrobial effect, high bioactivity, good cell growth and tissue, and increased fatigue power (Izman et al., 2012). The suitable metallic nanoparticles surface modification approaches help to make the material excellent in terms of its properties such as high formability, relatively low modulus, and good mechanical strength. Different types of surface modification approaches are summarized in Figure 5, which was generally used to enhance the biomedical applications by using metal derivatives nanoparticles.

Furthermore, the chemical, mechanical, oxidation, sol–gel, physical vapor deposition, and ion implantation are most popular methods used for surface modification (Kumar et al., 2020). The organic ligand is also considered as one of the good methods of surface modification to achieve the better outcomes. The organic groups are adequate to keep nanoparticles against accumulation; functional groups on nanoparticles surface may permit careful interaction of molecules with metal nanoparticles. The detailed working mechanisms of all these methods are explained previously in literature by many research groups such as Kango et al. (2013), Asri et al. (2017), Qi et al. (2019), Mozetič (2019), Oun et al. (2020), and Liu et al. (2020), respectively.

## Applications

There are different types of nanoparticles offering a potential platform for various biological-related applications. Nanoparticles have attracted significant research interest in biomedical applications. The incorporation of nanotechnology into biomedicine has opened new research possibilities. It has allowed valuable insights into molecular biology (Aziz et al., 2019). The nano-sized material has enabled many developments in biomedicine and other biological applications such as drug delivery, anticancer activity (Wani et al., 2016), gene delivery, fluorescent biological labels, protein detection, MRI contrast enhancement, probing of DNA, tissue engineering, phagokinetic studies, hyperthermia, and filtration of biological based molecular cells.

The nanoparticles nano size makes it appropriate to be used for biolabeling. The nano-sized nanoparticles can react with biomolecules at the surface level, as well as inside the cells, producing valuable signals and specific target for diagnosis and therapeutics process. Therefore, a large variety of nanoparticles with many possibilities of modification with other bio-based material have been explored for further biomedical testing. These new nanoparticles can have potential use in thermal ablation, imaging assays, drug delivery, radiotherapy enhancement, and gene delivery (Bushra et al., 2014). Some of the most important applications of different metallic nanoparticles are summarized as follows.

### Antimicrobial Agent

There are many reported antimicrobial agents that are found toxic to all living organisms. Therefore, to overcome this problem, different inorganic and metal-based antibacterial agents should have good thermal resistance, sustainability, and enhanced stability and synthesized under strict processing conditions (Rajawat and Qureshi, 2012; Hossain et al., 2015; Vijayakumari et al., 2019). Currently, Pt, Ag, Au, TiO_2_, ZnO, and so on, are the major metallic-based nanoparticles utilized as antibacterial agents. Metallic nanoparticles are mainly used as antimicrobial agents in biomedical applications because of their long-term stability and excellent biocompatibility.

**Figure 6:**
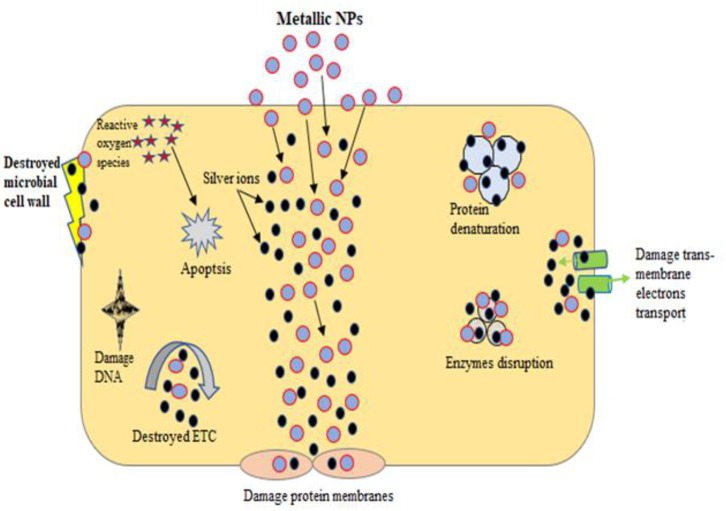
General mechanism of antimicrobial activities of metal-based nanoparticles.

Reported studies have proved that metal-based nanoparticles show biocidal activity against gram-negative and gram-positive bacteria (Franci et al., 2015; Chiriac et al., 2016; Rajeshkumar et al., 2016; Wang L. et al., 2017; Ovais et al., 2019). The antimicrobial effects of metal nanoparticles have been attributed to their nano size and high surface-to-volume ratio, which permits them to penetrate the bacterial membranes. The mechanisms of antibacterial effect of metallic nanoparticles are metal ion release, oxidative stress, and nonoxidative-based stress existing instantaneously as shown in Figure 6. Briefly, these nanoparticles serve only when nanoparticles interact with microbe's cell walls; several approaches for the contact of microbes nanoparticles were used such as van der Waals forces, electrostatic attraction, receptor/ligand, and hydrophobic interactions. After successful contact, metallic nanoparticles can pass through inner membranes, interact with metabolic paths, and induce variations in membrane morphology. Once nanoparticles interact with microbes inside cellular machinery, it acts as to prevent enzyme functions, disable proteins, electrolyte imbalance, induce oxidative stress, and change gene expression scale (Vijayakumari et al., 2019). However, excessive quantity of microbes produces barrier that resists antimicrobial mediators and microbes avoiding the resistant system by forming superantigens. The extracellular polymeric secretion also produces everlasting attachment of microbes.

Three most commonly known antimicrobial mechanisms of metallic nanoparticles are oxidative stress, nonoxidative, and dissolved metal ions. Reactive oxygen species (ROS)–induced oxidative stress is a very active approach of metallic nanoparticles action against microbes. The hydroxyl radical (OH), superoxide radical (O^−^), hydrogen peroxide, and single oxygen are related to ROS and considered as instant stress reactions. These can be condensed through endogenous antioxidants such as catalases and superoxide. In regular circumstances, equilibrium is sustained between clearance and generation of ROS in microbial cells. However, if excessive ROS production is carried out, the intracellular redox state is changed and supports oxidation process. Oxidative stress is a main contributor in fluctuating the microbial membrane penetrability and therefore can mutilation the cell membranes, respectively. Nano metallic ions stimulate oxygen and form ROS ions, as well as hydroxyl radicals, which can delay microbial production (Sangaonkar and Pawar, 2018). Furthermore, another commonly used mechanism is that in which metallic ions are slowly discharged from metal oxides in presence of aqueous medium and later absorbed by cell membranes, which leads to straight connections with functional sets of nucleic acids and proteins. The metallic nanoparticles interactions have extensive effects, which comprise cell physical variations and aberrant enzyme actions, and ultimately disturb usual physiological developments. The Ag and Pd nanoparticles were observed to have antimicrobial effects, which were ascribed to discharge the metallic ions into solution (Shaikh et al., 2019). (Zhao and Ashraf, 2015) studied the Ag nanoparticles mechanism as antimicrobial agent, in which Ag nanoparticles can go into biofilms and stop the biofilm growth through defeating gene expression. The tremendously nano dimensions particles are more valuable for achieving antimicrobial activities and fighting intracellular microbes. However, Slavin et al. (2017) found that the nano size of Ag (5–13 nm), Au (8–9 nm), ZnO (1–12 nm), and TiO_2_ (12–17 nm) nanoparticles carried out the excellent antimicrobial actions. The metallic nanoparticles produced from microbes have been used for antimicrobial purpose against pathogenic organisms. For example, biogenic-based Ag nanoparticles synthesized from *Bhargavaea indica, Brevibacterium frigoritolerans*, and *Sporosarcina koreensis* exhibited antimicrobial properties against *Salmonella enterica, Vibrio parahaemolyticus, B. cereus, Bacillus anthracis*, and *E. coli* (Singh et al., 2016).

Similarly, in case of Cu nanoparticles synthesized from *Sida acuta* showed good antimicrobial properties against *S. aureus, E. coli*, and *P. vulgaris*. The ZnO also exhibited antibacterial activities against *E. coli, S. aureus*, and *P. aeruginosa* (Sangaonkar and Pawar, 2018; Singh A. et al., 2018; Singh P. et al., 2018). Therefore, most metal-based nanoparticles show antimicrobial properties through many mechanisms. This limits the prospect of resistance against microorganisms. To make resistance toward metal-based nanoparticles, bacterial cells would have to undergo multiple instantaneous gene mutations, which are not feasible. Moreover, synthesizing environmentally benign metallic nanoparticles through green method is well documented to enhance the antimicrobial activities toward different pathogenic germs.

### Bioimaging Application

Because x-ray technology was used in medical imaging, several noninvasive methods have been developed and effectively functionalized in different areas of medical research such as drug discovery, drug delivery, clinical analysis, and investigation of cellular biology. Clinical imaging studies are carried out in many research areas and significantly contribute in the development of medicine. There are many modified methods of molecular bioimaging systems such as MRI, optical imaging, ultrasound imaging, positron emission tomography, and many others. These have been adapted for *in vitro* and *in vivo* biological treatment (Chen et al., 2018; Ferenz and Zhang, 2018; Pratiwi et al., 2019; Wang M. et al., 2019). In the study of metallic nanoparticles, an upgraded bioimaging technology such as MRI and optical imaging has been introduced.

Conde et al. (2012) stated that the noble metals such as gold, silver, and platinum nanoparticles can play valuable roles in instantaneous actuation and tracking due to the absorption of light from biological tissue at near-infrared wavelengths. Common noble metallic nanoparticles have been used in *in vivo* imaging therapies because of their powerful absorption of near-infrared radiation. Therefore, these nanoparticles can be considered as very active contrasting agents.

However, noble metallic nanomaterials can combine different multiple imaging modalities, which can be utilized to obtain significant information and provide synergistic benefits than any single type of bioimaging technique. The new trend of three-dimensional imaging might be achieved by using computed tomography because plane cross-sectional series of images are interweaved through the computer to produce three-dimensional images for clear results. The nanoparticles composites (FeO/Si core and Au shell composite) can be used in case of *in vivo* and composite materials that served as double contrast agents for computed tomography and MRI, which showed good computed tomography attenuation and gave better results of magnetic resonance in hepatoma (Gunko, 2016). Chauhan et al. (2019) proved that Gd-Au nanocomposite can be used for dynamic multimodal imaging as contrast agents.

Zhang W.-H. et al. (2016) studied the dye-loaded fluorescence imaging with Si nanoparticles in both *in vivo* and *in vitro* conditions. Si nanoparticles show excellent biocompatibility, less toxicity, excellent hydrophilicity, and potential optical transparency. Therefore, Si nanoparticles can serve as substrates for production of fluorescent probes, which is an important factor in bioimaging of cells. Li Z. et al. (2015) described the lanthanide-doped up-converting nanoparticles along with the bioimaging application. They suggested that these types of nanomaterials are convertible from low-energy to higher-energy state by using multiphoton processes. The authors elaborated the morphological chemistry of lanthanide-doped up-converting nanoparticles in biomedical applications such as imaging reagents, drug delivery, imaging guidable, and phototherapeutic factor.

Despite these significant progresses in bioimaging, there are still challenges that need to be explored. These low up-conversion productivity of lanthanide-based nanomaterials, problems related to the fabrication of subnano size (~10 nm) particles, and versatile progress at the commercial level are still unknown. Therefore, these challenges need to be addressed because the future research in bioimaging requires feasibility and reliability.

### Drug Delivery

**Figure 7:**
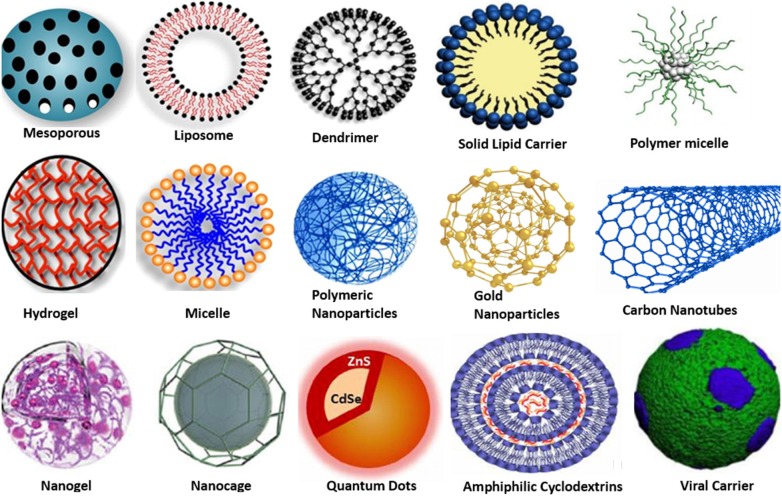
Different types of nanocarriers (nanoparticle) serve as drug delivery.

The encapsulation of drugs by using nanoparticles has been considered a promising and efficient method for drug delivery. Furthermore, the bioviability has been improved through incorporating different polymer-based nanoparticles (Nasimi and Haidari, 2013; Lu and Thum, 2019; Vissers et al., 2019). Metallic nanoparticle therapies lowered the therapeutic dosage, which improved the efficiency in treating cancerous cells. Chemotherapeutic agents injected into the patient's body show toxicity and poor compliance. It means that the delivery of therapeutic agent directly to the tumor cells is a critical challenge. Miller-Kleinhenz et al. (2018) studied the Dox-loaded FeO nanoparticles instantaneously associated with urokinase plasminogen activator receptor for active inhibition of cancerous cell. The targeted FeO-Dox nanoparticles caused powerful tumor development inhibition as compared with nontargeted. So, it is confirmed that the dual targeted FeO-Dox nanoparticles acted on appreciated stage for improved drug delivery approaches. Bogusz et al. (2014) described the hybrid-based nano gels produced from bismuth oxide–based quantum dots with polyvinyl alcohol for resolutions of heat sensing, bioimaging, and especially in drug delivery. Zhong et al. (2014) studied the MOF as potential drug carrier in the presence of Zn metal ions. The resultant MOF is [NH_2_(CH_3_)_2_]-[Zn_2_(HL)L0.5]n n(8DMF 5H_2_O), and it employed to load ibuprofen (IBU). The outcomes indicate that Zn ion can hold higher loading power of IBU, which is up to 50%, and show a longer discharged time within 96 h in replicated body fluid. Lee et al. (2011) summarized oleic acid–based FeO nanoparticles in oleic acid–conjugated chitosan (oleyl-chitosan) to observe the layer of nanoparticles in tumor tissues/cells by permeability and holding value under the *in vivo* condition for systematic practices by IR and magnetic resonance bioimaging mechanisms. In the *in vivo* assessments, both methods presented clear signal power and development in tumor cells/tissues through an advanced consequence after inoculation of cyanine-5–attached oleyl-chitosan nanoparticles intravenously. The bioimaging of target cells is achieved by using metal nanoparticles (Banerjee et al., 2016; Saini et al., 2016). The metallic nanoparticles react with biomolecules at both cell surfaces (inside and outside). It offers excellent transportation to therapeutics. The different metal nanoparticles and other nano size materials that have been introduced as drug delivery systems and diagnostics are shown in Figure 7. Nanoparticles also influence gene sequence in case of neoplastic cells, which results in cellular inflammation and protein expression such as cancer necrosis factors and so on. These growths of the cancerous cells depend on external and internal signals such as cytoskeletal function, pH, cellular proteins, and nuclear protein with cellular enzymatic sign, growing influence, free radicals, endoplasmic stress, and oxidative events. Kajani et al. (2016) stated that nanomedicine created innovative prospect in the upcoming progress of anticancer approaches. Traditional cancer treatments such as radiotherapy and chemotherapy are limited because of drug toxicity, drug resistance issue, and less specificity of targeted zone. Ag nanoparticles remove all these issues by reducing all drawbacks of aforementioned techniques and enhance the cancer treatment. Ag nanoparticles provide precise targeted drug delivery, and it has ability to cross living barriers. Green synthesis approaches of Ag nanoparticles also offer the delivery of cancer drug to tumor cells for betterment of cancer therapies.

Nanomedicine in injectable form is indispensable to target cancerous cells (Couvreur, 2013; Sanku et al., 2019; Sharma et al., 2019). On the other hand, these metal nanoparticles can simply conjugate with several mediators such as different types of antibodies, peptides, and RNA/DNA to target the various types of cells. These are eco-compatible to extend their flow *in vivo* for gene and medication delivery applications. They can convert light into other forms of energy, that is, heat; hence, it allows cancer cells to be targeted by thermal ablation. The antisense RNA is a valuable nucleic acid and targets the direction of RNA along with cancer cells (Young et al., 2016). Additionally, cell permeability and drug solubility impose severe challenge in the utilization of nanoparticles as therapy agents. The challenge can be overcome by improving the delivery system using nanotechnology. Generally, multiple nanoparticles are used in drug delivery methods. These have some common limitations that can be solved by changing the size, shape, layers, variety of applicable coating substance, solvents, surface potential, and different fabricating methods.

### Tissue Engineering

The nanoparticles received much attention recently in tissue engineering. Engineering and biological principles are used with practical substitutes of different types of damaged tissues. The nanoparticles that have low toxicity, tailorable properties, contrasting agent activities, targeted delivery potential, and accurate control over behavior make it suitable to use for developing/regenerating tissues (Corchero and Villaverde, 2009). The nanoparticles have been fabricated by using different materials (composites) to enhance its efficiency and role in tissue repairing. Several metallic nanoparticles were synthesized such as Ti, Au, Ag, and many others, which showed significant potential in this field. One of the possible uses of Au nanoparticles is in regenerative medicine, which is used in tissue swapping because of development of tumor cells (Lizundia et al., 2018; Bapat et al., 2019; Fathi-Achachelouei et al., 2019). Bioactive glass ceramic–based nanoparticles (BGC nanoparticles) are also very promising material and frequently used in bone tissue regeneration. Covarrubias et al. (2018) reported that the encapsulation of dense BGC nanoparticles into gelatin from chitosan polymer has encouraged the alkaline phosphate activities as compared to other mesoporous BGC nanoparticles. The result showed that the dense chitosan gelatin hydrogel with bioactive nanoparticles showed the highest rate of bone formation (80%) than others.

**Figure 8:**
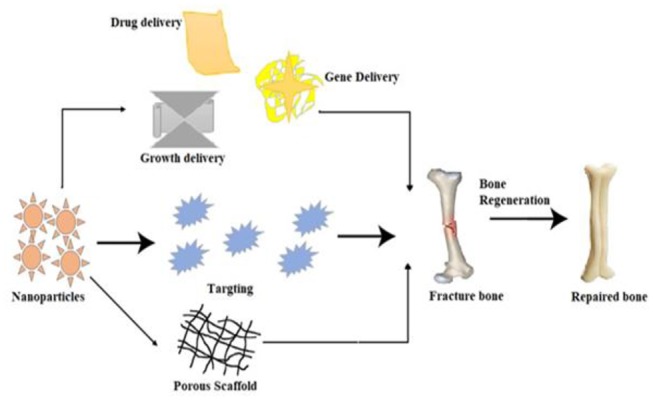
A schematic mechanism of the bone tissue engineering process by using nanoparticles and drug molecules.

Li et al. (2018) also studied the multifunctional poly–citrate-siloxane elastomer–based BGC nanoparticles and used it as an effective material in bone tissue regeneration. The titanium nanoparticles have also been used as bone repairing material. It is broadly used in the field of dentistry and orthopedics. It contains properties such as stronger fracture resistance, improved ductility, and effective weight-to-strength ratio. Unfortunately, they carried lower bioactivities, as it does not sustain cell adhesion and growth. Therefore, apatite coatings are considered preferable as bioactive materials. Hence, many methods were used to make the apatite coating around titanium. However, this coating also has some limitations such as non-uniformity in thickness, bad adhesion, and lower mechanical stability. Furthermore, stable absorbent structure is mandatory to support different nutrients via cell growth. A real bone is known as a valuable nanocomposite material, prepared by hydroxyapatite crystallites in different organic medium, which mostly consist of collagen. Bone is mechanically tough, so it can be resistant from any mechanical damage. Nanoscale mechanism that leads to such valuable amalgamation is still under consideration (Scott et al., 2013; Walmsley et al., 2015; Azizian et al., 2018; De-Witte et al., 2018). The general mechanism of bone tissue engineering is shown in Figure 8 to summarize the general concept used in bone engineering.

### Therapeutic Applications

**Figure 9:**
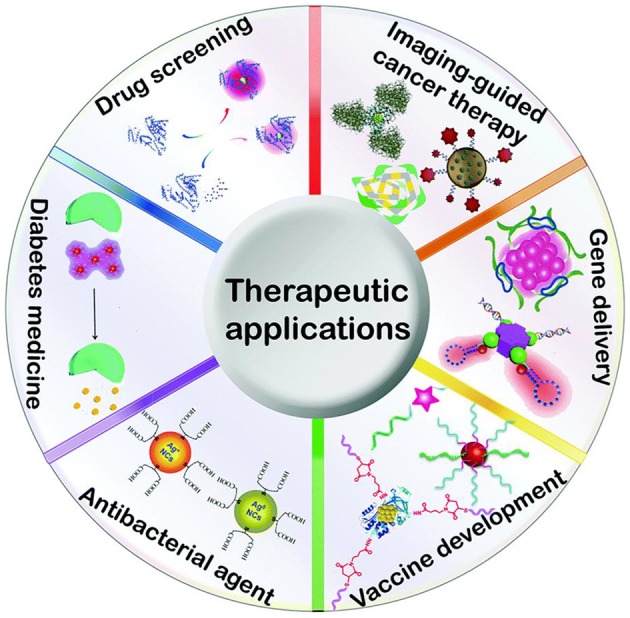
Schematic presentation of metal-based nanoclusters of therapeutic applications. Adapted from Tao et al. (2015), with permission from RSC.

The metallic nanoparticles or clusters have several significant and valuable features for therapeutic applications as shown in Figure 9. The study reported that metal nanoparticles have been designated as human immunodeficiency virus (HIV) preventive–therapeutic. In other studies, it has proven that silver nanoparticles can act directly on viral infection by interconnecting with glycoprotein (Liu and Chen, 2016; Saravanan et al., 2018). This type of interconnection prevents the different types of virial binding, which successfully reduces the HIV infection. Therefore, it is well known that metallic nanoparticles have been used as active antiviral agents to serve against respiratory syncytial, herpesvirus, and influenza.

In case of angiogenesis, the growth of original blood vessels takes place during usual growth and under disease conditions. It plays a vital role in several diseases such as arthritis and cancer. In stable conditions, the angiogenesis is strongly regulated among several proangiogenic evolution factors and a few antiangiogenic factors such as TSP-1 and platelet factor 4. Ramezani et al. (2008) studied the gold nanoparticles' effect on angiogenesis by using a mouse ear model inoculated with an adenoviral vector of vascular endothelial growth factor (VEGF). Chronic lymphocytic leukemia (CLL) is an irredeemable virus mainly due to apoptosis resistance. This is due to anti-VEGF antibodies, found in CLL-based cells. In CLL therapies, the Au nanoparticles were utilized to enhance performance of contracting agents. They were found to have good biocompatibility, large surface area, and high surface functionalization. The Au nanoparticles and the vascular endothelial growth factor antibodies were found to be involved. Their capacity to execute the CLL cells was measured.

Rheumatoid arthritis is a disease of the joints that arises when body immune system is not working effectively. It attacks the joints that connect bones. A group of researchers from the University of Wollongong, Australia, has assembled a new method for arthritic disease (Soni and Yadav, 2015; Ensafi et al., 2016; Zhang Q. et al., 2016). They introduced an antidrug that can be used through binding with Au nanoparticles, but it has some minor drawbacks. Another research has revealed that Au nanoparticles can attack macrophages and prevent inflammation. The size of Au nanoparticles is a very important factor in order to reduce the effect of toxicity, for example, a 50 nm or lesser size particle was more effective in enhancing the immunity of the cells without any side effects (Byeon et al., 2016; Dolati et al., 2016; Nogueira et al., 2016).

## Future Perspectives

The metal-based nanoparticles seem to have a commanding role in the twenty first century because of their vital role in nanomedicine and other biological applications. The nanoparticles can be fabricated by using several synthetic routes and effectively used in various nanomedical and biological applications. But there is still a need to prepare these nanoparticles on a commercial scale to reduce cost. The natural resources for the preparation of these nanoparticles should be sustainable, inexpensive, eco-friendly, and free from toxic chemical. It is important to produce monodispersed nanoparticles for future research. However, the mechanism to synthesize nanoparticles has not been completely explained at present. Therefore, future research should address the mechanism by which the shape and size of nanoparticles can be controlled. Another major challenge is the need to expand the use of nanoparticles in therapeutic applications and to reduce the level of toxicity. New strategies are being introduced with potential development in nanoscience to overcome the challenges by using noble metal nanoparticles. Prior to extensive use, impact on anthropological health factors needs to be considered.

Nanoscience can play a major role in modified biomedicine. It needs to be explored much more compressively in the future. The noble metal nanoparticles can serve as active agents in diagnosis and other therapeutic applications as they exhibit novel properties at atomic level, as well as at the supramolecular level. At present, there is a high demand for different types of nanomaterials and their composites in the health care, medical, and biological industries. Therefore, an emphasis must be made on the safety precautions to safeguard human health. In-depth studies on safety profiles are required specifically in the case of using metallic nanoparticles in health care. Future research should be directed to investigate the use of specific metal nanoparticles for individual applications.

The preparation of noble metal nanoparticles needs to be up-scaled from laboratory size to commercial medical size in the future. Metallic nanoparticles have been developed and are in an advanced state of testing in multidirectional applications. Currently, it is mainly used for the treatment of cancerous cells. Metal nanoparticles highlight their effectiveness as novel agents for future tumor therapeutic modalities. A question that needs to be answered is: Are the noble metal nanoparticles cytotoxic or biocompatible? Therefore, it is necessary to examine the nanoparticles interactions at laboratory scale and modify the nanoparticles for better biocompatibility with cells (cancerous) to remove injury to normal cells. Modern investigation should focus on the potential applications of nanoparticles at the commercial level, because in general, at present, all research is being conducted at a laboratory scale. Exploration at the commercial level may bring a revolution in the life of human beings.

## Conclusion

In this review, the importance of nanoparticles in biological and biomedicine is discussed. Metal-based nanoparticles have received much attention due to extensive medical testing and many other biological applications. Metal-based nanoparticles have potential to control antagonistic effects on different organs, different body tissues, and subcellular, cellular, and protein range because of their unique physicochemical properties. Furthermore, as the size of the nanoparticle decreases, some metal nanoparticles (Ag, Au, Zn, and Cu) can exhibit high toxicity, while on a comparatively bulk scale it is inactive. Metal–metal oxide or pure metal nanoparticles have been used as an appropriate substitute for antimicrobial studies. Antimicrobial nanoparticles could be used in therapeutics and in the production of medical devices with autodisinfection. It is highly possible that low-cost antimicrobial agents that are simple can be developed with metal, metal oxide, or metal oxide/metal–doped composites. It will serve as an alternative to traditional antibiotics. The noble metal nanoparticles and their composites are of great importance as they have been shown to have antimicrobial properties and used in drug delivery and also play an important role in many medical applications. Furthermore, the metal oxide nanoparticles have limited use due to their toxic effect, usually at high concentrations. It is proposed that ion doping, functionalization process, and conjugated polymer metal oxide nanoparticles can help to reduce the self-toxicity effect. Finally, it may be considered that metal, metal oxide, or composite nanoparticles with less toxicity will be used in the future for remediation of many dangerous infectious diseases and tumor cells.

## Author Contributions

AY and KU wrote the first version of manuscript. HA, TP, and AA drawn the figures and tables. MO, II, HQ, and MM provided suggestions and revised the manuscript.

## Conflict of Interest

The authors declare that the research was conducted in the absence of any commercial or financial relationships that could be construed as a potential conflict of interest.
